# Sudomotor dysfunction in patients with gluten neuropathy

**DOI:** 10.1007/s10072-021-05751-9

**Published:** 2021-11-17

**Authors:** Panagiotis Zis, Faiza Shafique, Ptolemaios G. Sarrigiannis, Artemios Artemiadis, Dasappaiah G. Rao, David S. Sanders, Marios Hadjivassiliou

**Affiliations:** 1 Medical School, University of Cyprus, Nicosia, Cyprus; 2 Medical School, University of Sheffield, Sheffield, UK; 3 Sheffield Teaching Hospitals NHS Foundation Trust, Sheffield, UK; 4 Department of Clinical Neurophysiology, Royal Devon and Exeter NHS Foundation Trust, Exeter, UK

**Keywords:** Gluten neuropathy, Small fiber, Sudomotor, SUDOSCAN, Autonomic

## Abstract

**Background and aim:**

Gluten neuropathy (GN) is a common neurological manifestation of gluten sensitivity (GS), characterized by serological evidence of GS, while other risk factors for developing neuropathy are absent. The degree of small fiber dysfunction in GN has not been studied in depth to date. Small fiber involvement may lead to pain, thermal perception abnormalities, and sweat gland dysfunction. Sudomotor innervation refers to the cholinergic innervation of the sympathetic nervous system through small fibers in the sweat glands. The aim of our study was to assess the sudomotor function of GN patients.

**Methods:**

Patients with GN were recruited. Clinical and neurophysiological data were obtained. HLA-DQ genotyping was performed. The skin electrochemical conductance (ESC) was measured with SUDOSCAN^TM^.

**Results:**

Thirty-two patients (25 males, mean age 69.5±10.2 years) were recruited. Thirteen patients (40.6%) had abnormal sudomotor function of the hands. Sixteen patients (50%) had abnormal sudomotor function of the feet. Twenty-one patients (65.6%) had abnormal sudomotor function of either the hands or feet.

Sudomotor dysfunction did not correlate with the type of neuropathy (length-dependent neuropathy or sensory ganglionopathy), gluten-free diet adherence, severity of neuropathy, and duration of disease or HLA-DQ genotype. No differences in the ESC were found between patients with painful and patients with painless GN.

**Conclusion:**

Sudomotor dysfunction affects two-thirds of patients with GN. The lack of correlation between pain and sudomotor dysfunction suggests different patterns of small fiber involvement in patients with GN.

## Introduction

Gluten neuropathy (GN), after gluten ataxia, is the commonest neurological manifestation of gluten sensitivity (GS) [1]. It is characterized by clinical and neurophysiological evidence of large fiber peripheral neuropathy (PN) and the presence of serological evidence of GS (presence of antigliadin, endomysial, and/or transglutaminase antibodies) in the absence of other risk factors for developing PN [1–3]. The commonest type of GN is the symmetrical “length-dependent” axonal PN followed by the sensory ganglionopathy (SG) [4].

GN is a relatively benign form of chronic PN, which shows very slow deterioration over time [5]. Despite the fact that it does not lead to severe disability, GN can lead to poor quality of life (QoL), mainly as a result of the presence of neuropathic pain [6, 7]. Being on a strict gluten-free diet improves the overall QoL and reduces dramatically the odds of suffering from peripheral neuropathic pain (PNP) [8]. PNP is present in about 60% of patients suffering from GN, a figure also seen in PN of other etiologies [9–15]. PNP is a result of small fiber involvement [16]. Apart from pain, small fiber dysfunction may also lead to autonomic failure. Sudomotor innervation is a term referring to the cholinergic innervation of the sympathetic nervous system through small fibers in the sweat glands.

The aim of this cross-sectional study was to assess the sudomotor function of patients with GN and investigate any determinants contributing to sudomotor dysfunction.

## Methods

### Inclusion criteria

A diagnosis of GN was made based on clinical symptoms of peripheral neuropathy, and its type was confirmed electrophysiologically [17, 18]. GS was confirmed serologically. All patients were extensively investigated for the presence of other risk factors for developing neuropathy based on our published protocol [2]. All patients were adults and provided written informed consent.

### Measures

The Overall Neuropathy Limitations Scale (ONLS) was used to assess the clinical severity of the neuropathy [19].

We considered as being on a successful gluten-free diet (GFD) patients who have achieved a negative serology at the time of recruitment.

We used the visual analogue scale, ranging from 0 to 10, to determine the PNP intensity. Only patients reporting pain intensity of equal to or greater than 4 were considered to suffer from a painful neuropathy.

### HLA genotyping

The HLA-DQ typing was performed using the polymerase chain reaction utilizing sequence-specific primers.

### Sudomotor assessment

Sudomotor function was assessed via SUDOSCAN^TM^. Patients were asked to place their hands and feet on the devices’ electrodes for 3 min. An electrical stimulus (<4 V) was automatically generated. The electrochemical skin conductance (ESC), measured in micro-Siemens (μS), was recorded. Sudomotor function was graded according to the values of ESC with normal being 70–100μS, moderately reduced 50–70 μS, and severely reduced (0–50 μS )for the feet and for the hands normal being 60–100μS, moderately reduced 40–60 μS, and severely reduced 0–40 μS [20]. The overall ESC for the hands was calculated by adding the ESC from the left and the ESC from the right hand and by dividing by 2. The overall ESC for the feet was calculated by adding the ESC from the left and the ESC from the right foot and by dividing by 2.

### Statistical analyses

Comparisons between groups were made using Mann-Whitney *U* test for non-normally distributed and chi-square test or Fischer’s exact test for categorical data. For comparison of the ESC means of our study population with published ESC means of other population, we used MedCalc. Spearman correlation coefficients were calculated for numerical by numerical associations. *p* values lower than 0.05 were considered to be significant.

### Data availability

All anonymized data can be shared following a reasonable request.

### Ethical issues

## Results

### Study population

Thirty-two patients (25 males, mean age 69.5±10.2 years) with gluten neuropathy were recruited. Four patients had coeliac disease, while 28 had GS. Eight patients were diagnosed with SG and 24 with length-dependent PN. These two groups did not differ significantly regarding age, gender, or presence of CD.

Our study population had a mean duration of symptoms of 15.7±7.4 years and a mean duration since neuropathy diagnosis of 12.6±6.0 years. The mean ONLS arm score was 1.2±0.8, the mean ONLS leg score was 1.8±1.2, and the mean total ONLS score was 2.9±1.8.

Seventeen patients had a DQ2 or DQ8 genotype.

None of our patients had evidence of dermatitis or other skin disorders that could have affected the electrochemical skin conductance test.

Table 1 summarizes the study sample characteristics.

**Table 1:** Study sample characteristics (*N*=32)

Characteristic	*N* (%), mean ± SD
Males	25 (78.1)
Age (years old)	69.5 ± 10.2
Disease	
*Coeliac disease*	4 (12.5)
*Gluten sensitivity*	28 (87.5)
Neuropathy	
*Length-dependent*	24 (75)
*Sensory ganglionopathy*	8 (25)
Symptom duration (years)	15.7 ± 7.4
Duration since neuropathy diagnosis (years)	12.6 ± 6.0
Total ONSL score	2.9 ± 1.8
*Arm ONSL score*	1.2 ± 0.8
*Leg ONSL score*	1.8 ± 1.2
DQ2/DQ8 positive	17 (53.1)
Presence of pain	23 (71.9)
GFD	16 (50)

*GFD* gluten-free diet patient, *ONSL* Overall Neuropathy Limitations Scale, *SD* standard deviation

### Assessment of electrochemical skin conductance test

Thirteen patients (40.6%) had abnormal sudomotor function of the hands. Sixteen patients (50%) had abnormal sudomotor function of the feet. Twenty-one patients (65.6%) had abnormal sudomotor function of either the hands and/or feet. Among those, 8 patients had abnormal ESC on both the hands and feet; 3 had severely reduced ESC on both the hands and feet, 1 had moderately reduced ESC in the feet but severely in the hands, 2 had moderately reduced ESC in the hands but severely in the feet, and 2 had moderately reduced ESC on both the hands and feet.

Table 2 summarizes the degree of sudomotor dysfunction for the total population.

**Table 2:** Degree of sudomotor dysfunction based on electrochemical skin conductance (ESC) (*N*=32).

Characteristic	N (%), mean (SD)
Feet ESC in μS (SD)	62.7 (21.5)
Hands ESC in μS (SD)^1^	61.0 (16.8)
Normal ESC	11 (34.4)
Abnormal ESC, hands and feet	8 (25)
Abnormal ESC, hands or feet	13 (40.6)
Abnormal ESC, hands only	5 (15.6)
Abnormal ESC, feet only	8 (25)
Abnormal ESC, hands^1^	13 (40.6)
*Moderately reduced*	9 (28.1)
*Severely reduced*	4 (12.5)
Abnormal ESC, feet	16 (50.0)
*Moderately reduced*	10 (31.3)
*Severely reduced*	6 (18.7)

*SD* standard deviation

^1^For one patient, the overall electrochemical skin conductance value on the hands was assessed only by the left hand because of an industrial traumatic injury of the right arm. Sudomotor function was graded according to the values of ESC with normal being 70–100μS, moderately reduced 50–70 μS, and severely reduced (0–50 μS ) for the feet, and for hands normal being 60–100μS, moderately reduced 40–60 μS, and severely reduced 0–40 μS

### Determinants of sudomotor dysfunction

Comparison between groups showed that sudomotor function, including the degree of asymmetry between the two sides, was similarly impaired in patients with SG and patients with length-dependent PN. Patients being on a successful GFD (*n*=16) showed similar sudomotor function with patients being on either a partial GFD (*n*=16) or on normal diet (*n*=6).

No differences in the ESC were found between patients with painful GN and patients with painless GN (Table 3). Among patients with painful GN, pain intensity did not correlate significantly with the ESC. The severity of neuropathy, determined by the ONLS score, did not correlate with the sudomotor function (Spearman’s rho −0.039, *p*=0.834 for the hands, Spearman’s rho −0.219, *p*=0.229 for the feet). Furthermore, neither the duration of the neuropathic symptoms nor the duration since neuropathy diagnosis correlated with sudomotor function of either the hands (Spearman’s rho −0.277 *p*=0.131 and Spearman’s rho −0.240 *p*=0.194, respectively) or the feet (Spearman’s rho −0.022 *p*=0.906 and Spearman’s rho −0.103 *p*=0.576, respectively) . HLA-DQ alleles as well as DQ2 and DQ8 genotypes were checked for their effect on ESC revealing no significant results (data not shown).

**Table 3:** Demographic, clinical, and electrochemical characteristics of patients with GN and with and without pain. *GN* gluten neuropathy, *PN* peripheral neuropathy, *ONLS* Overall Neuropathy Limitations Scale, *SD* standard deviation.

	Painful GN (***n***=24)	Painless GN (***n***=8)	***p*** value
***Demographics***			
Age, in years (SD)	70.0 (10.3)	68.0 (10.4)	0.521
Male gender (%)	19 (79.2)	6 (75.0)	0.577
***Clinical characteristics***			
Length-dependent PN (%) Sensory ganglionopathy (%)	4 (16.7) 20 (83.3)	4 (50.0) 4 (50.0)	0.082
ONLS arm score (SD)	1.2 (0.8)	1.1 (1.0)	0.255
ONLS leg score (SD)	1.8 (1.2)	1.8 (1.3)	0.337
Total ONLS score (SD)	2.9 (1.7)	2.9 (2.0)	0.176
Duration of symptoms in years (SD)	16.3 (7.6)	14.0 (6.8)	0.925
Duration since diagnosis in years (SD)	13.1 (5.2)	11.1 (8.3)	0.692
***Electrochemical skin conductance (ESC)***			
Feet, mean ESC, in μS (SD)	61.1 (21.3)	67.3 (22.7)	0.821
Hands, mean ESC, in μS (SD)	61.0 (17.1)	60.6 (18.3)	0.192

## Discussion

This cross-sectional study demonstrates that two out of three patients with GN have sudomotor dysfunction, which indicates sympathetic nervous system impairment. ESC was measured with a validated tool, SUDOSCAN^TM^, a non-invasive technology that assesses the C-small sympathetic nerve fibers of the sweat glands [21] via determination of chloride conductance across the skin [21].

Symptomatic autonomic dysfunction in the context of gluten sensitivity (GS) and coeliac disease (CD) has been reported previously [22]. In their case series, Gibbons and Freeman described 4 patients with CD that presented with symptoms such as orthostatic lightheadedness, syncopal, or presyncopal episodes and palpitations. Autonomic testing which included expiratory to inspiratory heart rate variability, Valsalva manoeuver with blood pressure and heart rate analysis, and tilt table testing confirmed autonomic dysfunction. Moreover, Penny et al. showed that the prevalence of GS and CD among patients with postural tachycardia syndrome (PoTS), a condition associated with dysautonomia, is significantly higher compared to the general population [23].

To our knowledge, this study is the first to assess autonomic function in patients with GN. An interesting finding was that sudomotor function did not correlate with the presence of pain, suggesting different patterns of small fiber involvement in patients with GN. Contrary to the positive effect that GFD has on pain [8], being on a strict GFD in this cohort appears to have no effect, though this finding should be interpreted with caution as this is not an interventional prospective study but a cross-sectional one. Another interpretation might be that sympathetic small fiber involvement may be irreversible in these patients. We have made the observation that some patients with painful small fiber neuropathy are often left with significant neuropathic pain despite strict adherence to GFD. Our analyses showed that there are no particular determinants for the presence of sudomotor dysfunction, including an absence of an HLA association.

In a recent study where SUDOSCAN^TM^ was used, Krieger et al. assessed sudomotor function in a cohort of 27 patients with diabetic PN (DPN) [24]. The mean ESC in their patients with DPN did not differ significantly from our study population (feet, 64.4±16.5 versus 62.7±21.5, *p*=0.738; hands, 58.2±18.2 versus 60.9±17.1, *p*=0.560). This may suggest that the degree of sudomotor dysfunction in patients with large fiber PN does not differ across different etiologies, but this remains to be confirmed.

Previous studies have shown that a strict GFD, where an elimination of the circulating antibodies occur, is linked to a favorable outcome after at least 12 months on diet [25]. Moreover, we have previously shown that patients with GN being on a GFD have significantly less odds for having neuropathic pain [8]. In many cases, GFD alone may be adequate for the successful management of neuropathic pain, even without the use of medication. In our cohort, however, sudomotor function did not differ between patients on a strict GFD compared to patients on a gluten-containing diet. However, this was a cross-sectional study, and therefore, an etiological link between gluten consumption and sudomotor dysfunction cannot be established.

Our results should be interpreted with some caution given the limitations of our design. Firstly, our sample consisted only of 32 patients, which—although is a relatively small number—is a well-defined population that we have been regularly following up for many years. As our cohort comprised of users of one specialized service, these results may not be generalizable to other settings. Moreover, we have only used one tool (SUDOSCAN^TM^) to assess sudomotor function. Although SUDOSCAN^TM^ is a validated tool for assessing sudomotor function, our results would have been stronger if were complemented by the use of other sudomotor function measurements. Finally, we used the manufacturers’ normal values to determine abnormalities in the SUDOSCAN^TM^ measurements. Having had a control group of subjects without neuropathy would have also strengthened our study.

In conclusion, sudomotor dysfunction is very common in patients with GN. The natural history of this involvement could be studied in a prospective larger study.
